# A combined view of B-cell epitope features in antigens

**DOI:** 10.6026/97320630015530

**Published:** 2019-08-15

**Authors:** Nayem Zobayer, ABM Aowlad Hossain, Md.Asadur Rahman

**Affiliations:** 1 Department of Biomedical Engineering, Khulna University of Engineering and Technology, Khulna 9203, Bangladesh; 2 Department of Electronics and Communication Engineering, Khulna University of Engineering and Technology, Khulna 9203, Bangladesh

**Keywords:** B-cell, epitope, antigens, combined

## Abstract

B-cell epitope mapping is a promising approach to identify therapeutics and vaccine candidates in antigenic proteins. We used MATLAB programming to view in combination different features such as beta turn region, surface accessibility, antigenicity and hydrophilicity in an antigen sequence to help predict a discontinuous, conformational B-cell epitope. We analyzed, grouped, compared, matched and superposed these features for a combined visualization using MATLAB programming for identifying and illustrating a potential B-cell epitope region in an antigen protein. This protocol finds application in the design and development of an effective B cell epitope candidate.

## Background

Prediction of potential B cell epitopes is of importance in the development of efficient therapeutic antibodies and target specific vaccines [1]. The main reason for epitope prediction is to substitute an antigen in the therapeutic antibody production, sero-diagnosis and immunization [2]. The precise prediction of B cell epitopes holds a basic thumb rule for development of antibody therapeutics [3], peptide-based vaccines [3, 4] and immuno-diagnostic tool [5]. Available epitope mapping (both structural and functional approach) methods are relatively expensive, time consuming, laborious, and sometimes fail to detect all potential epitopes [6]. Structure based epitope mapping models predicts protein structure using amino acid residues in direct contact with an antibody and sometimes with no information on the binding strength of thes amino acids. So, the prerequisite for successful B cell epitope mapping is property wise identification and characterization of amino acids for the identification of antigenic portion of proteins.

There are some B cell epitope prediction tools available, among them ABCpred [7], BCPREDS [8, 9], BepiPred [10] and Bcepred [11] are used. These tools are not standalone to predict B cell epitopes. In order to identify potential B cell epitopes propensity scale-based tools are often used in several models [12-15]. Different prediction tools are frequently used to reach a conclusion on identifying an effective B cell epitope. Available tools offer low sensitivity, specificity and accuracy. Hence, there is ample space for further improvement. The main objective is to design discontinuous conformational B cell epitopes using sequence data.

Parameters such as antigenicity, surface accessibility, flexibility, beta turn, and hydrophobicity are used to define a discontinuous, conformational B cell epitope. Therefore, it is important to classify potential regions and non-significant regions of each scale. Successful identification and classification of protein's antigenic, surface accessible, hydrophilic, flexible and beta turn regions are of importance to select suitable B-cell epitopes. Hence, we describe different parameters and classify them on different scales to help in the identification of potential B cell epitopes.

## Methodology

We used MATLAB programming to classify the different features of a protein sequence to help predict a potential B cell epitope from a protein or a group of protein sequences. A protein sequence (FASTA format) with the accession number AAY57281.1 from the UniProtKB database was used as a test sequence in this study.

## Beta-turn regions:

**Figure 1:**
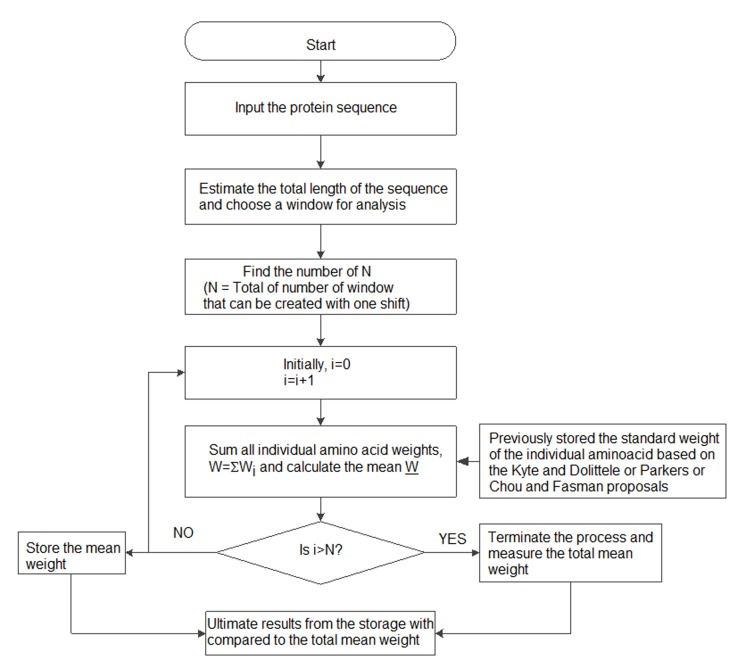
A block diagram for grouping B cell epitope features in a protein antigen.

**Figure 2:**
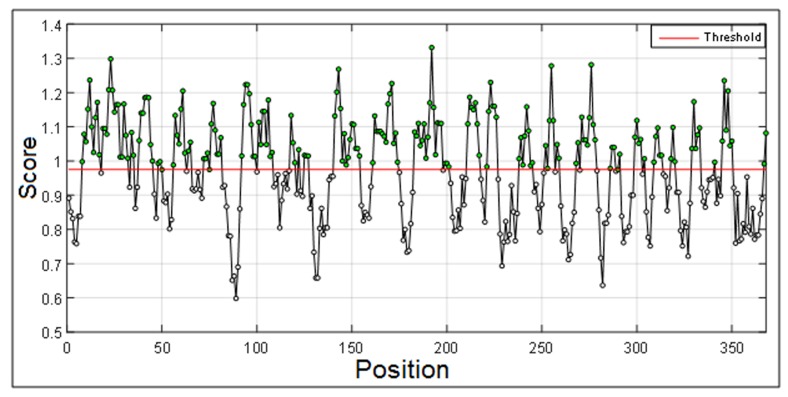
Graphical presentation of beta turn region in a protein antigen

Secondary structure elements in a protein are usually alpha helix, beta turn regions, and coil-coil regions. Beta turn region is relevant to epitope design. Hence, an algorithm that efficiently classifies beta turn regions from alpha helix and coil-coil regions is needed as shown in a flowchart (Figure 1). Chou and Fasman [16] secondary structure prediction scale for proteins was used in this study. We used the Chou and Fasman method [16] incorporated into the MATLAB interface for generating a plot (Figure 2) for beta turn regions with window size from i = 0 to i > N.

## Hydropathicity:

**Figure 3:**
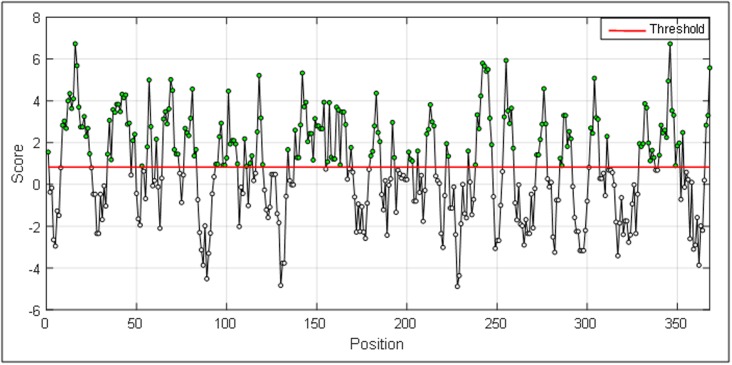
Hydropathicity in a protein antigen.

A hydropathicity scale for amino acids was proposed by Parker and colleagues [17]. This scale for amino acids was used to identify potential hydrophilic regions in the query protein for generating a plot (Figure 3) with window size from i = 0 to i > N.

## Surface accessibility:

**Figure 4:**
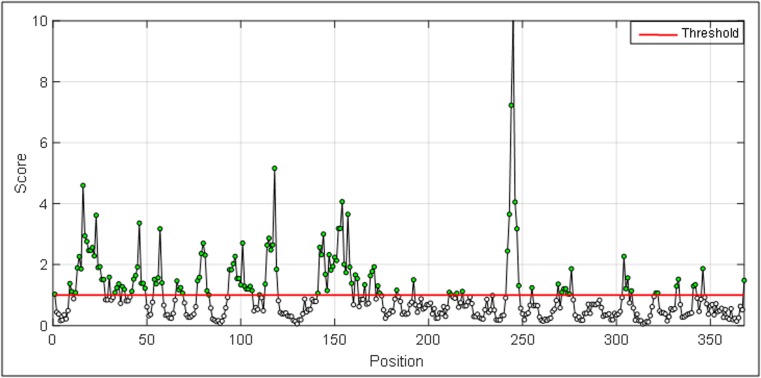
Graphical presentation of surface accessibility in a protein antigen.

The empirical amino acid accessible surface probabilities according to Janin and colleagues [18] which are fractional probabilities (0.26 to 0.97) determined for an amino acid found on the surface of a protein is used. A surface residue is defined as one with >20 Å of water-accessible surface. The most surface accessible area in a protein (Figure 1) was determined with these fractional surface probabilities for amino acids, which a surface probability after calculating normalized surface accessible values for amino acids and a plot was generated as shown in Figure 4.

## Antigenicity prediction:

**Figure 5:**
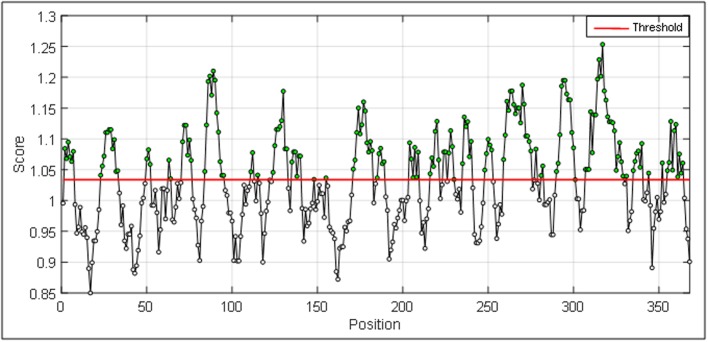
Graphical presentation of antigenicity in a protein antigen.

Kolaskar and Tongaokar developed a semi-empirical method which utilizes physicochemical properties of amino acid residues and their probabilities or frequencies of occurrence in experimentally known segmental epitopes to predict antigenic determinants on proteins [19]. Application of this method to a large number of proteins has shown by the Kolaskar and Tongaonkar that the method can predict antigenic determinants with about 75% accuracy which is better than most of the known methods. Kolaskar and Tongaonkar method was used to calculate antigenicity in the query protein for generating a plot (Figure 5) with window size from i = 0 to i > N.

## Results and Discussion:

It is of interest to develop a standalone algorithm with graphical representation for B cell epitope prediction from FASTA format protein sequence. The features of B cell epitope are hydrophilicity, surface accessibility, beta turns, exposed surface, polarity and antigenic properties of amino acids. These properties of polypeptides chains have been correlated with the location of the continuous and discontinuous conformational epitopes. This has led to a search for empirical rules that would allow the position of continuous epitopes to be predicted from certain features of the protein sequence. All calculations are based on propensity scales for each of the 20 amino acids. Each scale consists of 20 values assigned to each of the amino acid residues on the basis of their relative propensity to possess the property described by the scale.

The outputs are illustrated as graphical representations (Figure 2 to Figure 5). The Y-axes depicts the correspondent score (averaged in the specified window) for each residue and the X-axes correspond to the residue positions in the sequence. The larger score for the residues is interpreted as that the residue with a higher probability to be part of a potential epitope (those residues are above the red line threshold on the graphs). However, the presented method does not predict the epitopes per se, either linear or discontinuous; they might only guide to further explore the protein regions on being genuine B cell epitopes. Here, we separately built graphs for beta turn, hydropathicity, surface accessibility and antigenicity in a protein antigen. Higher scores in graphs denote higher probabilities of being a B cell epitope. Finally, all the graphs were superimposed onto each other to identify the region(s) of the protein which is highly antigenic, flexible, situated on the beta turn, hydrophilic and surface accessible, simultaneously. This region will be considered as potential B cell epitope and can be used for further vaccine development.

**Figure 6:**
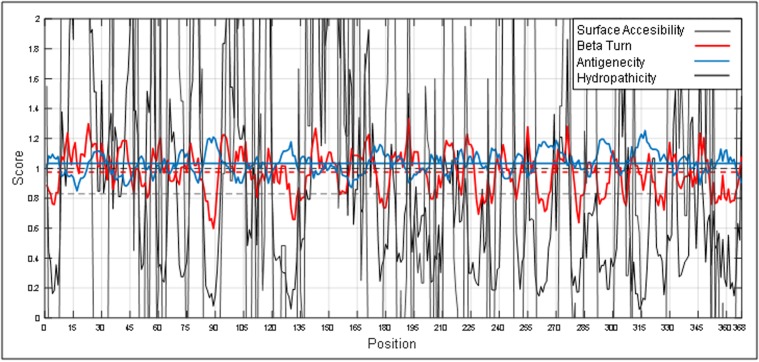
Combined view of beta turn, hydropathicity, surface accessibility and antigenicity in a protein antigen to define a potential B cell epitope.

**Figure 7:**
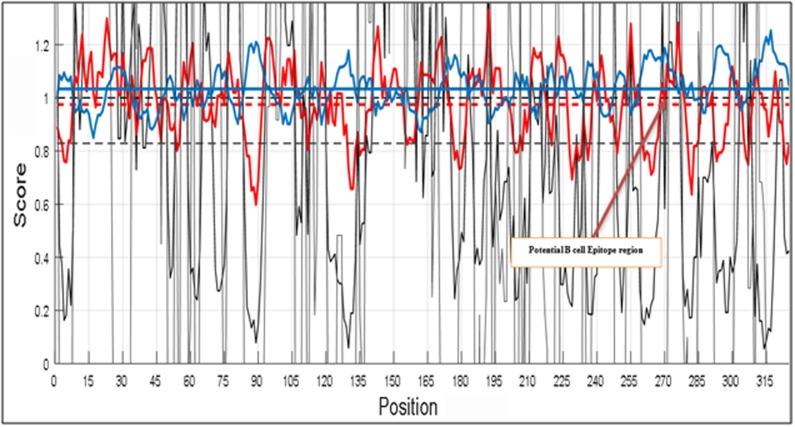
Partial zoomed in image for showing a potential B cell epitope determined by combining different protein features.

Figure 2 illustrates regions having beta turn regions. The spikes above the threshold are probable beta turn regions. Most hydrophilic regions in a protein are shown (Figure 3). The regions above the red threshold line are hydrophilic regions and the line beneath the threshold is a hydrophobic region. We thus distinguish hydrophilic regions from the hydrophobic regions. It is a prerequisite to identify beta turn regions and hydrophilic regions in a protein antigen to define epitopes. We also classified the most surface accessible regions in a protein while defining B cell epitopes (Figure 4). Finally, we classified the most antigenic regions of a protein according to the literature data as shown in Figure 5. Lastly, all four features are grouped into a single graph to define a potential B cell epitope region (272-280) in the protein antigen with accession AAY57281.1 (Figure 6). A zoomed version of Figure 6 is given in Figure 7 for more clarity.

B cell identification in the protein antigen with accession AAY57281.1 was previously reported [13] by applying traditional methods of identification, which involved using ten different tools [13] to reach a conclusion. Nevertheless, the study concluded that, GDRIPDEKN (12-20) and PHVPEYSSS (273-281), two 9-mer peptides could be the most effective B-cell epitopes of AAY57281.1. The results presented in this report matches known data for a potential B cell epitope (272-280) with more precision and sensitivity. This will reduce time and cost in finding a potential B cell epitope in a protein antigen.

## Conclusion

Identification of B cell epitope in a protein antigen will be relatively easy, simple, less time consuming and more precise in coming years. We show the combined view of features such as beta turn, surface accessibility, antigenicity and hydrophilicity in an antigen protein sequence to define a potential B cell epitope. The proposed protocol is promising pending cross validation and testing with an updated dataset.

## Availability:

MATLAB scripts are made available at https://www.mathworks.com/matlabcentral/fileexchange/72322-protein-analyzing-tools
